# Isolation and assessment of characterization of porcine epidemic diarrhea virus GD1 strain from China

**DOI:** 10.3389/fvets.2026.1912420

**Published:** 2026-08-07

**Authors:** Shuxia Shi, Qiuxia Wang, Xufan Cheng, Xiaobing Wei, Renfeng Li, Feifei Tao, Zhuan Yang, Xiumei Li, Xuejian Ren, Changzhong Liu, Haiyan Gao

**Affiliations:** 1 College of Animal Science and Veterinary Medicine, Henan Institute of Science and Technology, Xinxiang, China; 2 Henan International Joint Laboratory of Animal Health Breeding and Disease Prevention and Control, Xinxiang, China; 3 Hexu (Zhengzhou) Biotechnology Co., Ltd, Zhengzhou, China

**Keywords:** pathogenicity, phylogenetic analysis, porcine epidemic diarrhea virus, spike protein, virus isolation

## Abstract

Porcine epidemic diarrhea virus (PEDV) is an important pathogen in swine, causing severe economic losses to the global swine industry. As a coronavirus, PEDV is prone to mutations and recombination. Therefore, characterizing circulating strains is essential for disease control. In this study, a PEDV strain (GD1) was isolated from diarrheic piglets in Guangdong Province, China, and identified by RT-PCR and immunofluorescence. Pathogenicity was evaluated in piglet challenge experiment. The results showed that GD1 strain could be stably passaged in Vero cells, forming syncytia. Phylogenetic analysis based on the complete genome and spike (S) gene both placed GD1 strain within the GIIc genotype. Amino acid sequence alignment of the S protein revealed multiple amino acid mutations in the neutralizing epitopes COE, SE16, and SS6 of GD1 strain compared with the classical vaccine strain CV777. Recombination analysis suggested that GD1 may be a novel recombination strain originating from strains SD2021 and GDS28, with the recombination breakpoint located in the ORF1a region. Pathogenicity assessment demonstrated that GD1 is highly virulent, inducing severe watery diarrhea and vomiting in newborn piglets within 24 h post-inoculation, and causing histopathological lesions characterized by intestinal villous atrophy and shedding. Immunohistochemistry confirmed that PEDV antigen was predominantly distributed in the villous epithelial cells of the small intestine. The successful isolation and characterization of the GD1 strain provide important viral resources for further research on the genetic evolution of PEDV and the development of matched vaccines.

## 1 Introduction

Porcine epidemic diarrhea (PED) is an acute and highly contagious disease caused by the porcine epidemic diarrhea virus (PEDV), characterized by watery diarrhea, vomiting, and dehydration (1). Pigs of all ages are susceptible to PEDV infection; however, clinical symptoms and mortality rates vary significantly with age. Notably, infection in newborn piglets within 7 days of age can lead to a mortality rate approaching 100% under field conditions in immunologically naive herds, particularly in the absence of maternal immunity (2, 3), resulting in substantial economic losses to the swine industry. Since its initial identification in Europe, PEDV has proliferated at an accelerated rate in Asia, where it has become pervasive. The first PED case in China was identified in 1984. A large-scale PED outbreak occurred in China in late 2010, which was attributed to variant PEDV strains and caused significant economic losses to the domestic swine industry (4). In 2013, variant PEDV strains spread widely in the United States, and subsequently, outbreaks caused by variant strains were reported in several provinces in China (5–7). Currently, PEDV remains prevalent in China. Moreover, PEDV strains from China and South Korea exhibit higher recombination frequencies and evolutionary rates compared to strains from other regions, suggesting that circulating strains in Asia may possess more complex genetic backgrounds and stronger environmental adaptability (8).

PEDV belongs to the Alphacoronavirus genus in the family of Coronaviridae, along with transmissible gastroenteritis coronavirus (TGEV), porcine respiratory coronavirus (PRCoV), and swine acute diarrhea syndrome coronavirus (SADS-CoV). These viruses are enveloped, single-stranded positive-sense RNA viruses with genomes of approximately 28 kb (9, 10). The genome contains a 5′-cap structure, a 3′-poly(A) tail, and seven open reading frames (ORFs). These ORFs encode nonstructural proteins, structural proteins, and an accessory protein, arranged in the following order: Non-structural proteins (Nsp1-16), spike protein (S), accessory protein (ORF3), envelope protein (E), membrane protein (M), and nucleocapsid protein (N) (11). The nonstructural proteins are primarily involved in viral replication, transcription, and regulation, and play a critical role in immune evasion (12). The ORF3 protein encodes an ion channel protein and is associated with PEDV virulence (13, 14). The E protein and M protein interact to participate in virion assembly and budding (15). The N protein is the major and highly conserved structural component of the viral particle; it encapsulates the viral RNA to form the viral nucleocapsid and participates in viral replication, transcription, and translation processes, and exhibits strong immunogenicity (16). The S protein of PEDV is a transmembrane glycoprotein located on the surface of the virion, containing several critical neutralizing epitopes, including COE (aa 499–638), SE16 (aa 722–731), SS2 (aa 748–755), SS6 (aa 764–771), and 2C10 (aa 1,368–1,374). These epitopes are primary targets of virus-neutralizing antibodies (17) and play pivotal roles in virus-host interactions and immune evasion mechanisms (18, 19).

Global PEDV strains can be classified into two major groups based on mutations in the S gene: group GI (classical strains), group GII (variant strains), and S INDEL genotypes. The GI group includes subtypes GIa and GIb, while the GII group comprises subtypes GIIa, GIIb, and GIIc (20). Since its introduction into China in 2013, the GII subtype has rapidly become the predominant circulating strain, characterized by strong transmissibility and high pathogenicity (21). In a systematic analysis of 1,109 PEDV strains circulating in China, GIIa accounted for 35.62%, GIIb for 25.70%, and GIIc for 26.06% of the total subtypes (22). Recent report showed that 66.67% of strains isolated in a separate study across six provinces in China were categorized as subtype GIIc (23). Moreover, another study indicates that the GIIc subtype has significantly increased since 2018, reaching 92.3% in 2024 (24). These results indicated that GIIc has become predominant in China in recent years. During 2022–2024, the GIIc lineage was the exclusive dominant lineage, accounting for 93.6% (29/31) of sequenced strains in Guangdong Province (25). Recombination is a major evolutionary driver of PEDV genetic diversity, especially in the GIIc lineage, which is characterized by frequent recombination events and antigenic drift, contributing substantially to the emergence of novel variants, undermining conventional vaccine efficacy and complicating disease control efforts (26–28). Recombination events have been identified across multiple genomic regions, including ORF1a (27), ORF1b (29), and S gene (30), which can compromise vaccine-induced immunity and thereby pose significant challenges to existing prevention and control systems (31).

Guangdong Province is one of China’s major pig-producing regions and has been identified as a high-incidence area for PEDV (22). Continuous efforts in the isolation, identification and pathogenicity studies of PEDV are of great significance for elucidating its epidemiological patterns, developing better-matched vaccines and formulating effective prevention and control strategies. Previous reports have documented recombination events in PEDV strains circulating in Guangdong, with breakpoints predominantly identified in the S gene (32). In this study, a PEDV GIIc strain, designated as GD1, was successfully isolated from clinical samples collected from a pig farm in Guangdong Province that was experiencing an acute diarrhea outbreak. The strain was characterized and its pathogenicity was assessed. Notably, this is the first report of a recombination event occurring in the ORF1a region in a PEDV strain circulating in Guangdong Province, providing a scientific basis for epidemiological surveillance and the development of effective control strategies.

## 2 Materials and methods

### 2.1 Clinical samples

Intestinal tissue samples were randomly collected from 6 piglets with diarrhea at a commercial pig farm in Guangdong Province, China, in 2024. The neonatal piglets (3–7 days old) in the affected farm suffered from acute watery diarrhea. Six minced intestinal tissues were homogenized using a tissue grinder, suspended in sterile PBS, freeze–thawed three times, and centrifuged at 2,000 rpm for 5 min at 4 °C. The supernatants were collected and stored at −80 °C for further analysis.

### 2.2 RT-PCR identification of PEDV

The total RNA was extracted with the TRIzol reagent (TaKaRa, Dalian, China), and then cDNA was synthesized using a commercial reverse transcription kit (TaKaRa, Dalian, China) according to the manufacturer’s instructions. The cDNA was used as template to amplify the PEDV N gene. Specific primers targeting the PEDV N gene were designed based on the conserved region (Table 1). The corresponding genes of TGEV, PDCoV, PRRSV, CSFV, and PRCoV were simultaneously amplified to identify the pathogen in infected piglets. The reaction system was in 25 μL, consisting of 12.5 μL of Premix Taq™ (TaKaRa, Dalian, China), 1 μL each of forward and reverse primers (10 μmol/L), 2 μL of cDNA template, and sterile water to adjust the volume. The amplification was performed under conditions as follows: pre-denaturation at 98 °C for 1 min; 30 cycles of denaturation at 98 °C for 15 s, annealing at 55 °C for 30 s, and extension at 72 °C for 70 s; followed by a final extension at 72 °C for 10 min. PCR products were detected by 1% agarose gel electrophoresis. The purified PCR products were sequenced and compared with the sequences in GenBank.

**Table 1:** Name and sequences of primers used in the identification of pathogen.

Name of the primer	Sequence (5′ → 3′)	Product sizes (bp)
PEDV-F	TATGCCCCTCCTAGGGTTAC	1,216
PEDV-R	CAACAGCTGTGTCCCATTCC	
TGEV-F	TTGTCAACACACAAGGGCAAGC	426
TGEV-R	CAAGTCCAAAAGTGCGATCACC	
PDCoV-F	GCAAGTTCAAGCAGAGCAAG	672
PDCoV-R	ACGGTGTTAAGAGCCTCAGA	
PRRSV-F	GAGTTTCAGCGGAACAATGG	451
PRRSV-R	GCCGTTGACCGTAGTGGAG	
CSFV-F	CGAGATGCTAYGTGGACGAGG	293
CSFV-R	TCAGTGTTGAYTGTGGGTGTACC	
PRoV-F	CCCCGGTATTGAATATACCACAGT	335
PRoV-R	TTTCTGTTGGCCACCCTTTAGT	

### 2.3 Cells and antibodies

Vero cells were maintained and passaged in Dulbecco’s modified Eagle’s medium supplemented with 10% fetal bovine serum (Gibco, Thermo Fisher Scientific, Waltham, MA, USA). Rabbit anti-PEDV N protein antiserums were prepared using a standard protocol.

### 2.4 Virus isolation

The processed supernatant of intestinal tissue was decontaminated using a 0.22 μm filter and dispensed for virus isolation. Vero cells were cultured until they reached approximately 80% confluence. The cell monolayer was rinsed twice and inoculated with 0.5 mL of the processed supernatant of intestinal tissue. After incubating for 1 h at 37 °C in a 5% CO_2_ incubator, the inoculum was discarded and replaced with virus maintenance medium (containing 7 μg/mL trypsin). Cells were blind passaged and observed daily for cytopathic effect (CPE). When CPE exceeded 80%, the cell culture was harvested. Following three additional freeze–thaw cycles, blind passage was continued until stable CPE were consistently observed. Subsequently, the TCID_50_ of virus was calculated using the Reed-Muench method. Genomic sequencing, immunofluorescence assays, and animal challenge experiments were performed using virus at passage 20 (P20), at which stable CPE was consistently observed.

### 2.5 Indirect immunofluorescence assay

The isolated virus was inoculated onto Vero cells in a 24-well plate, with mock-infected cells serving as the control. All cultures were maintained at 37 °C in a 5% CO_2_ incubator until distinct CPE developed. After discarding the medium, cells were rinsed with PBS and fixed with 4% paraformaldehyde for 25 min at room temperature. Following three washes with PBS, cells were permeabilized with 0.1% Triton X-100 (Beyotime Biotechnology, Shanghai, China) at room temperature for 15 min and washed again three times with PBS. After blocking with 5% BSA (Solarbio, Beijing, China) for 1 h and another three washes, cells were incubated overnight at 4 °C with rabbit anti-PEDV N protein antiserum (1:100). Then the cells were washed and incubated with a 1:500 dilution of FITC-labeled goat anti-rabbit IgG (Beyotime Biotechnology, Shanghai, China) at 37 °C for 1 h in the dark. Following washes, nuclei were stained with DAPI (Beyotime Biotechnology, Shanghai, China) for 5 min at room temperature in the dark. After final washes, samples were observed under a fluorescence microscope (Nikon, Tokyo, Japan).

### 2.6 Whole-genome amplification and sequencing

Based on the conserved region sequence of PEDV, 17 pairs of overlapping primers were designed (Table 2) on the basis of CV777 strain (AF353511) available in GenBank. The complete viral genome sequence was amplified by RT-PCR using LA Taq DNA polymerase (TaKaRa, Dalian, China) according to the manufacturer’s instructions. The PCR products were gel-purified and sequenced at Sangon Biotech (Shanghai, China). The obtained results were assembled into a complete genome using SeqMan in DNASTAR 11 (DNAStar Inc., Madison, WI, USA) and the final sequence was deposited in the GenBank database.^1^

**Table 2:** Name and sequences of primers used in whole genome amplification.

Name of the primer	Sequence (5′-3′)	Product size (bp)
1F	GTTCCGTCGCCTTCTACA	1,319
1R	AGGTCACCAAGCTTACGTAT	
2F	AATGTTGTTTTGTCTGAGCC	2,450
2R	GGTGCAGAGGAGTCTACTTC	
3F	ACGTTGCCATTGAGGTTC	2,565
3R	GCGTGACACGTTCAATAG	
4F	GTTAACGGTAGGCGTGTGAT	2,273
4R	GCAATGAAATCACGTACAAG	
5F	TCATCGCTATGATGTCCTCTT	2,803
5R	GCACGCAAACATAGGTGTAAC	
6F	CAAATTCTTGGCCATACCTCG	2,428
6R	TGCAAACGTCATTCTCAAGT	
7F	GTTAAGTGGGAGTTCGATGG	2,223
7R	CACGTTCTTTGCCACTAATA	
8F	ACATCCTGCCCACTATGAC	2,765
8R	GGCAACATAATTCTGGCT	
9F	ATTGTTGTGGTGGATGAAG	2,900
9R	CCGTAATCTTCAAGTCCGTA	
10F	CTATGAAGCCGAACGTCC	2,734
10R	TGGCAAAGATGGCTAAATGA	
11F	GAAGGCTTCTTTCCCTAT	2,967
11R	GAAGGCTTCTTTCCCTAT	
12F	AAGGTGACGGAGTTTAGTTG	2,909
12R	GCTGAGTTGTAATGCTGACTCTAT	
13F	TTTTGACCTTGACGATGG	2,813
13R	ACAGCATCCAAAGACAAGTT	
14F	TTGAGTGGCTCAACCGAG	1,288
14R	CAAGAGGCCAAAGTATCCAT	
15F	TTGTTGCGCACTTATTGGC	1,540
15R	TGAGGTCCTGTTCCGAGGTAG	
16F	TATGGCTTGCATCACTCT	2014
16R	GGATCTTTAATTACTCGTGC	
17F	GAGCTAGCGGACTCTTAC	547
17R	GGATCTTTAATTACTCGTGC	

### 2.7 Phylogenetic analysis and recombination analysis

To perform phylogenetic analysis, reference sequences of 37 PEDV strains were downloaded from the GenBank database (Table 3). Multiple sequence alignment of the complete genome and S gene sequences, including the isolate GD1, was performed using the Clustal W algorithm in MEGA 11.0 software (Mega Limited, Auckland, New Zealand). Based on this alignment, a phylogenetic tree was constructed using the Neighbor-Joining method, with branch support assessed by 1,000 bootstrap replications. Recombination analysis of the isolated strain was conducted with RDP v4.101 software (RDP Project, East Lansing, MI, USA) with nine detection algorithms (RDP, GENECONV, BootScan, MaxChi, Chimaera, SiScan, 3Seq, LARD and Phylpro), and detected recombination events were further evaluated using SimPlot++ 1.3 software (Université du Québec à Montréal, Montreal, QC, Canada), and only signals consistently identified by both detection methods were considered reliable.

**Table 3:** PEDV strains used in the sequence alignment and phylogenetic analysis.

Reference strains	Accession	Countries	Year
CV777	AF353511	Belgium	1978
attenuated DR13	JQ023162	Korea	2003
LZC	EF185992	China	2006
JS2008	KC109141	China	2008
SM98	GU937797	Korea	1998
SD-M	JX560761	China	2012
DR13	JQ023161	Korea	2011
BJ-2011-1	JN825712	China	2011
SD2014	KX064280	China	2014
CH/HNAY/2015	KR809885	China	2015
PC22A	KY499262	USA	2015
AH2012	KC210145	China	2012
KNU-1305	KJ662670	Korea	2013
KGS-1/JPN/2013	LC063814	Japan	2013
NIG-1/JPN/2014	LC063830	Japan	2014
GDS28	MH726372	China	2012
AJ1102	JX188454	China	2012
LW/L	MK392335	China	2019
CH/GX/PEDV/1401/2016	MZ364310	China	2016
CH/GX/PEDV/2373/2018	MZ364314	China	2018
CHSD2014	KX791060	China	2014
CH/SCZG/2017	MH061337	China	2017
ZL29	KU847996	China	2015
KNU-1406-1	KM403155	Korea	2014
KCH-2/JPN/2014	LC063845	Japan	2014
USA/Indiana12.83/2013	KJ645635	USA	2013
USA/Iowa106/2013	KJ645695	USA	2013
OH851	KJ399978	USA	2013
USA/Ohio126/2014	KJ645702	USA	2014
GDGZ-2020	ON964511	China	2020
HB/HEBEU/2020	MW413556	China	2020
SD2020	OL762456	China	2020
SC2021	OL411879	China	2021
SD2021	OL762459	China	2021
HK2021	OL762457	China	2021
HN2021	OK584017	China	2021
CH/GDJM2023	PQ316090	China	2023

### 2.8 Pathogenicity evaluation

Six 3-day-old healthy newborn Large White piglets, with an average birth weight of 1.4 kg, diagnosed as negative for PEDV, transmissible gastroenteritis virus (TGEV), porcine reproductive and respiratory syndrome (PRRSV), porcine deltacoronavirus (PDCoV), classical swine fever virus (CSFV), and PRCoV by RT-PCR, were purchased from Henan Liaoyuan Pig Breeding Improvement Co., Ltd. They were randomly allocated into two groups: a mock control group (*n* = 3) and a PEDV-challenged group (*n* = 3). All piglets were artificially fed with fresh liquid milk instead of breast milk every 4 h. Following the method of Sun et al. (33), after a two-day acclimation period, the piglets in the experimental group were orally inoculated with 3 mL of GD1 strain virus suspension (10^4.3^ TCID_50_/mL), while the piglets in the control group received 3 mL of virus-free DMEM orally at the same time. Then, clinical symptoms of the piglets were observed daily. When challenged piglets exhibited severe diarrhea, emaciation, and instability of gait, they were euthanized using ear intravenous injection of sodium pentobarbital (150 mg/kg body weight) in a separate compartment and underwent necropsy. The duodenum, jejunum, and ileum were collected and fixed in 4% paraformaldehyde for subsequent histopathological examination and immunohistochemistry analysis. The study was concluded at 48 h post-infection when all challenged piglets reached the humane endpoint.

### 2.9 Histopathological examination and IHC staining

The intestinal tissues fixed in 4% paraformaldehyde were embedded in paraffin, and sectioned into 5 μm thick using a microtome. The sections were mounted on slides, then deparaffinized with xylene, rehydrated in decreasing gradient ethanol, and stained with hematoxylin and eosin (H&E) to perform histopathological analysis. For immunohistochemistry, rehydrated sections were washed three times with PBS. Antigen retrieval was performed using 0.01 M sodium citrate buffer. Sections were incubated with 3% H_2_O_2_ for 10 min to quench endogenous peroxidase activity and then washed thrice with PBS. After blocking with 5% BSA at room temperature for 1 h, sections were incubated overnight at 4 °C (10–12 h) in a humidified chamber with rabbit anti-PEDV N protein polyclonal antibody diluted 1:100 in PBS. Following three PBS washes, sections were incubated with HRP conjugated goat anti-rabbit IgG (Beyotime, Biotechnology, Shanghai, China) at 37 °C for 1 h and washed again three times with PBS. Immunoreactivity was visualized using DAB (Beyotime, Biotechnology, Shanghai, China) substrate for 3–10 min, and the reaction was stopped by rinsing with distilled water. Finally, sections were counterstained with hematoxylin, dehydrated through increasing gradient ethanol, mounted, and examined under a light microscope.

## 3 Results

### 3.1 Virus isolation and identification

**Figure 1:**
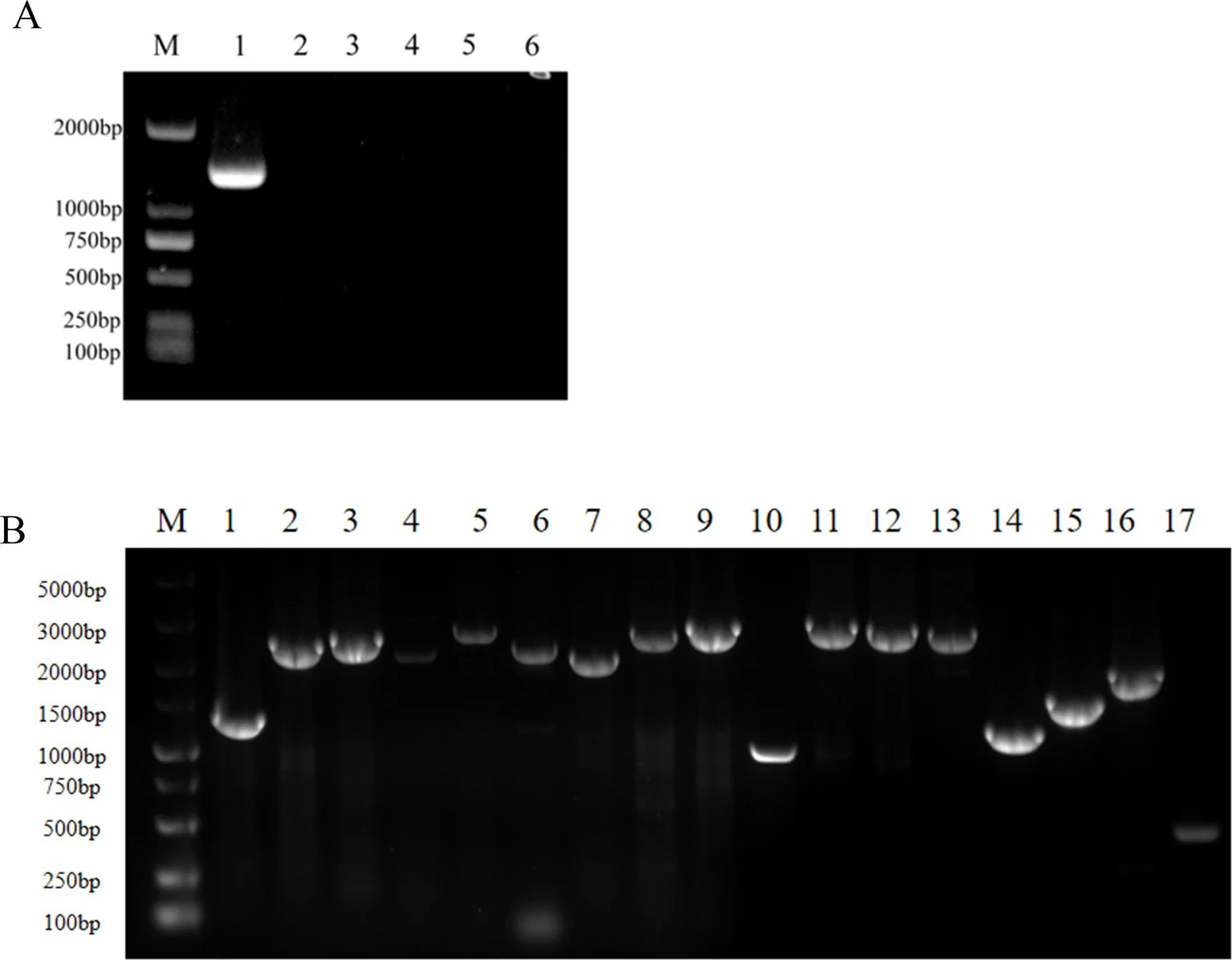
PCR identification and whole genome amplification of PEDV. **(A)** PCR identification of PEDV isolate. Lane M: DL2000 DNA marker; Lane 1–6: amplification results of PEDV, TGEV, PDCoV, PRRSV, CSFV, and PRCoV, respectively. **(B)** Whole genome amplification of PEDV isolate. Lane M: DL5000 DNA marker; Lane 1–17: amplification results of 17 fragments.

Total RNA from intestinal tissues of diseased piglets was used to amplify and identify PEDV N gene using specific primers, and a target band of 1,216 bp was observed in test lane, while no bands were observed in the control lane including TGEV, PDCoV, PRRSV, CSFV, and PRCoV (Figure 1A). Then, the target band was purified and sent for sequencing. The results from sequencing and blasting showed that the sequences amplified from the positive samples were consistent, representing the same PEDV strain, which was designated as strain GD1. To analyze the genetic characteristics of GD1 strain, the whole genome was amplified by conventional RT-PCR using 17 pairs of overlap primers based on the genome sequence of the PEDV CV777 strain (AF353511), and the expected sizes bands (Figure 1B) were sent for sequencing and assembling. The assembled complete genomic sequence of the PEDV GD1 strain comprises 28,043 nucleotides (nt) and has been submitted to GenBank with accession number PX146708.

**Figure 2:**
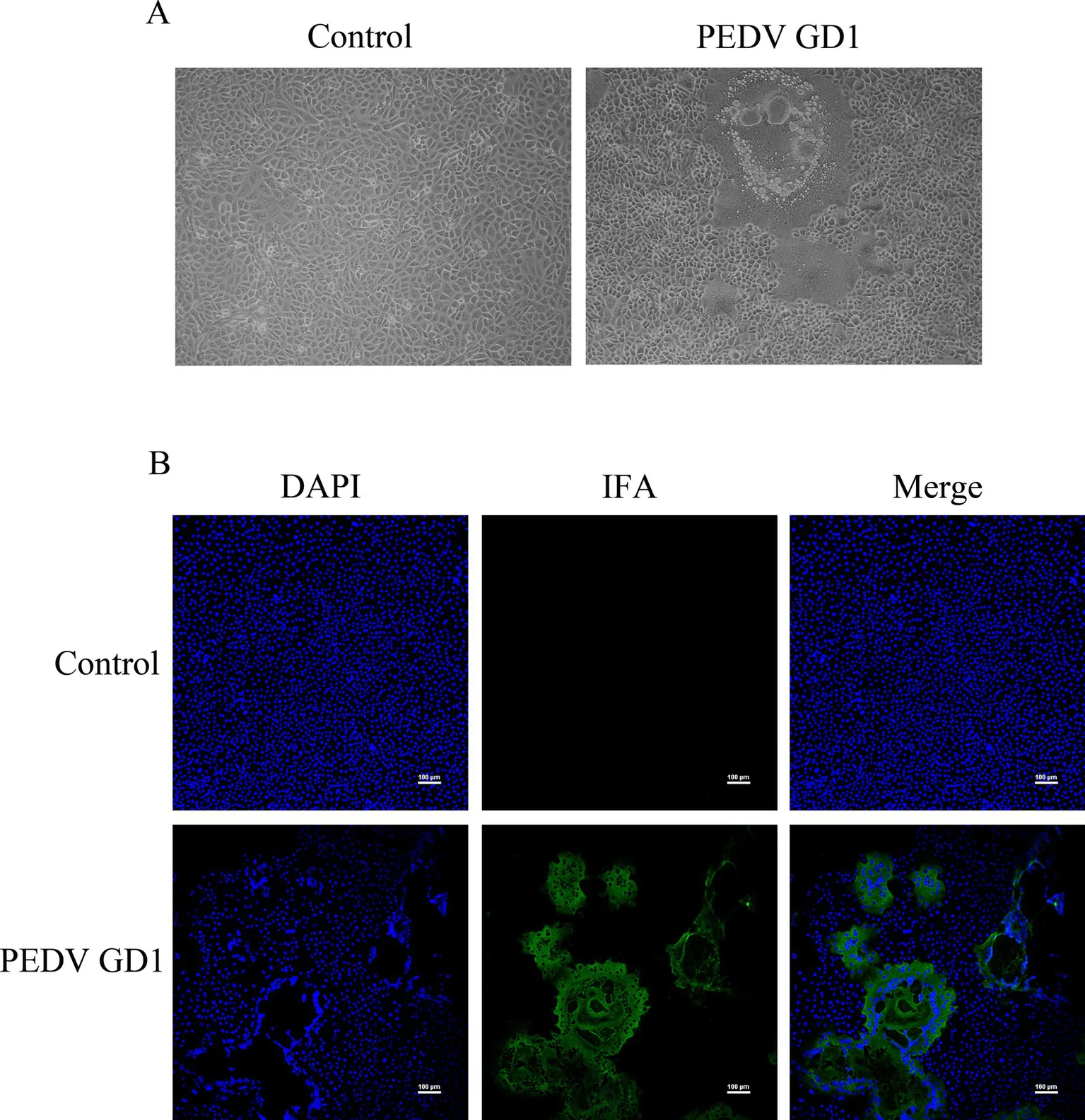
Identification and characterization of PEDV GD1 strain on Vero cells. **(A)** Cytopathic effect of PEDV GD1 strain on Vero cells at 36 hpi (100×). **(B)** Detection of PEDV GD1 strain in Vero cells by immunofluorescence staining at 36 hpi (100×).

Moreover, one positive sample was randomly selected and inoculated onto Vero cells for virus isolation and propagation. Following successive passages, distinct cytopathic effects were observed, characterized by cell rounding, cell fusion and syncytium formation (Figure 2A). Immunofluorescence assay was conducted and specific green fluorescence within the cells was observed at the sites of cytopathic changes (Figure 2B). The viral titer of GD1 reached a peak of 10^4.3^ TCID_50_/mL at 36 hpi. These results further confirmed that PEDV was successfully isolated.

### 3.2 Phylogenetic analysis

**Figure 3:**
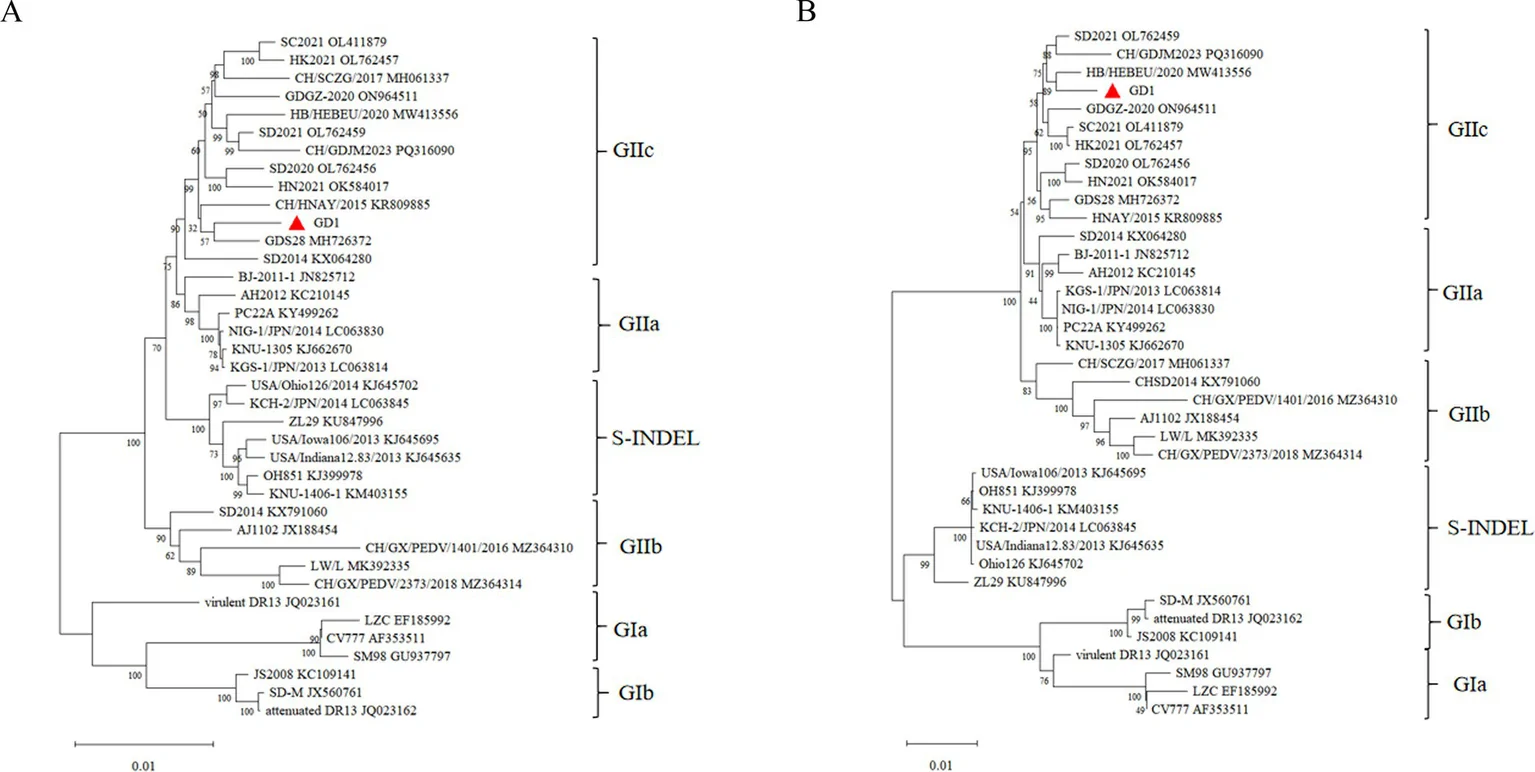
Phylogenetic analysis of PEDV GD1 strain. **(A)** Phylogenetic analysis based on the whole genome of PEDV GD1 strain and PEDV reference strains. **(B)** Phylogenetic analysis based on the S gene nucleotide of PEDV GD1 strain and PEDV reference strains. The tree was constructed using the neighbor-joining method in MEGA 11.0 software. Numbers above the branches are bootstrap values computed from 1,000 replications. The filled triangle indicates the PEDV GD1 strain identified in this study.

The genotype and genetic variation of GD1 strain were compared with those of reference strains representing different genotypes based on the sequence of the complete genome, S gene, N gene, and ORF3 gene, respectively. As shown in Table 4, the nucleotide sequence homology of complete genome among GD1 and GI, GIIa, GIIb, GIIc, S-INDEL subgroup strains were 96.3–98.4%, 98.7–98.8%, 97.5–98.4%, 98.8–99.2%, and 98.3–98.4%, respectively. The homology analysis based on the S gene among GD1 and the GI, GIIa, GIIb, GIIc, S-INDEL subgroup strains was 92.9–95.8%, 98.3–98.6%, 96.8–98.4%, 98.3–99.1%, and 95.6–95.8%, respectively. The phylogenetic tree based on the complete genome sequences showed that the GD1 strain clustered within the separate branch of GIIc subgroup strains, indicating a close genetic relationship with strains such as GDS28 and CH/HNAY/2015. In contrast, it was placed on a distinct branch from classical GI strains like CV777, suggesting a more distant relationship (Figure 3A). The phylogenetic tree based on the S gene yielded consistent results, confirming that the PEDV GD1 strain belongs to the GIIc genotype (Figure 3B).

**Table 4:** Analysis of the nucleotide sequence homology.

Primer	Percentage of nucleotide identity (%)				
	GI	GIIa	GIIb	GIIc	S-INDEL
Whole-genome	96.3–97.4	98.7–98.8	97.5–98.4	98.8–99.2	98.3–98.4
S	92.9–94.3	98.3–98.6	96.8–98.4	98.3–99.1	95.6–95.8
N	94.7–96.3	98.0–99.0	96.0–96.8	98.6–99.1	98.2–98.7
ORF3	95.3–98.2	98.1–99.7	95.3–96.7	97.2–99.9	99.0–99.7

### 3.3 Analysis of the S protein amino acid sequence

**Figure 4:**
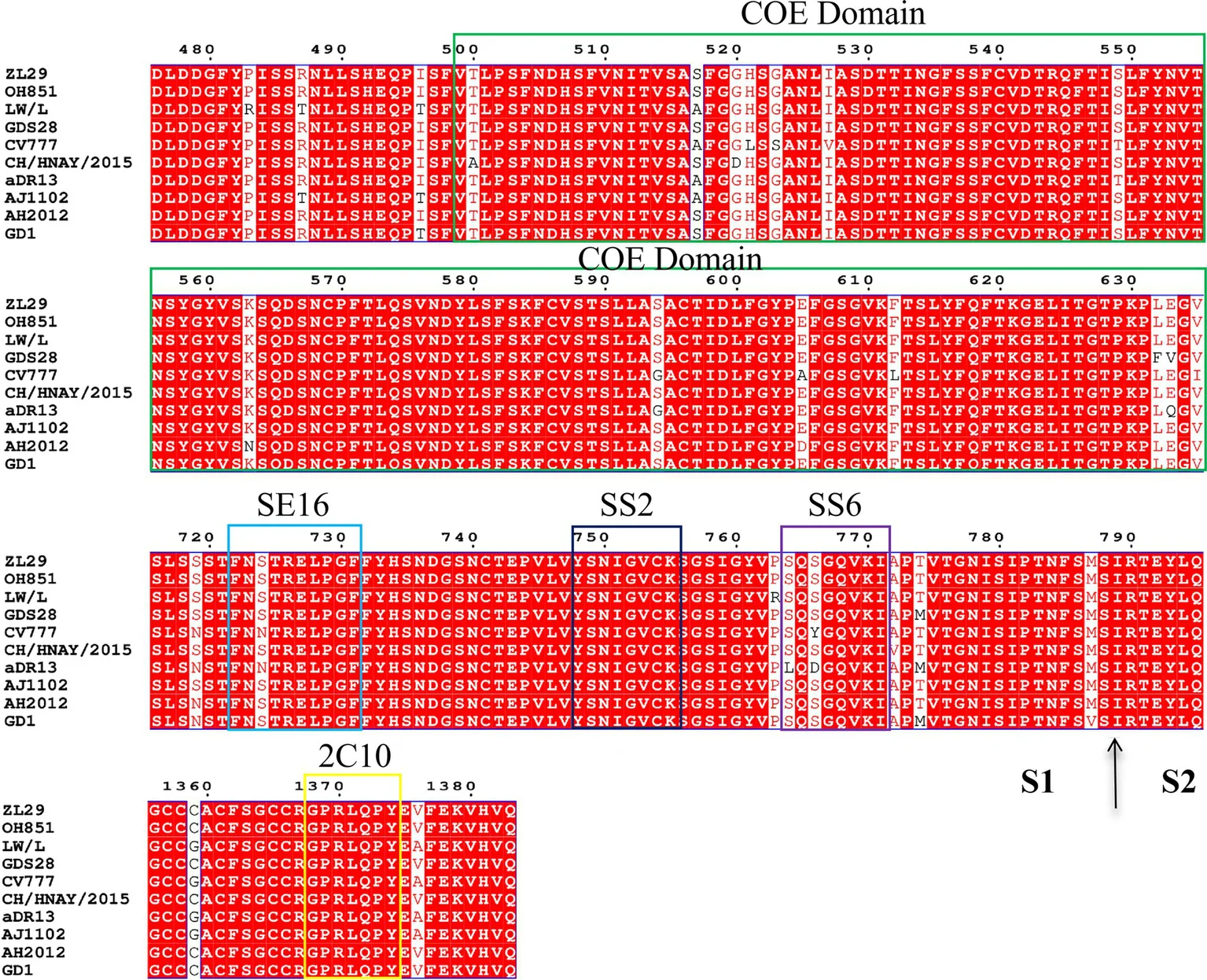
Genetic variation analysis of S protein from PEDV GD1 strain in five neutralizing epitopes among representative strains.

The sequence of S protein was compared with those of reference strains representing different genetic subgroups (CV777, ZL29, OH851, LW/L, GDS28, CH/HNAY/2015, attenuated DR13, AJ1102, AH2012) to analyze the genetic variations of PEDV GD1 strain. Amino acid sequence alignment analysis revealed multiple amino acid mutations in five neutralizing epitopes (COE, SE16, SS2, SS6, and 2C10) of S protein amino acid sequence (Figure 4). The epitopes SS2 and 2C10 were highly conserved, while more amino acid mutations were present within the COE, SE16, and SS6 epitopes. Compared to the classical strain CV777, the GD1 isolate harbored nine amino acid mutations (A517S, L521H, S523G, V527I, T549S, G594S, A605E, L612F, and I635V) within the COE domain, and one mutation each in the SE16 (N724S) and SS6 (Y766S) domains. When compared with the AJ1102 strain, a representative strain of GIIb subgroup, the GD1 isolate exhibited a single amino acid mutation (A517S) in the COE domain. Furthermore, this isolate possessed seven unique amino acid mutations (H106Y, P226L, M787V, A883S, L995M, L1041S, and V1268I) relative to other strains (Table 5).

**Table 5:** Amino acid differences of S protein among representative strains.

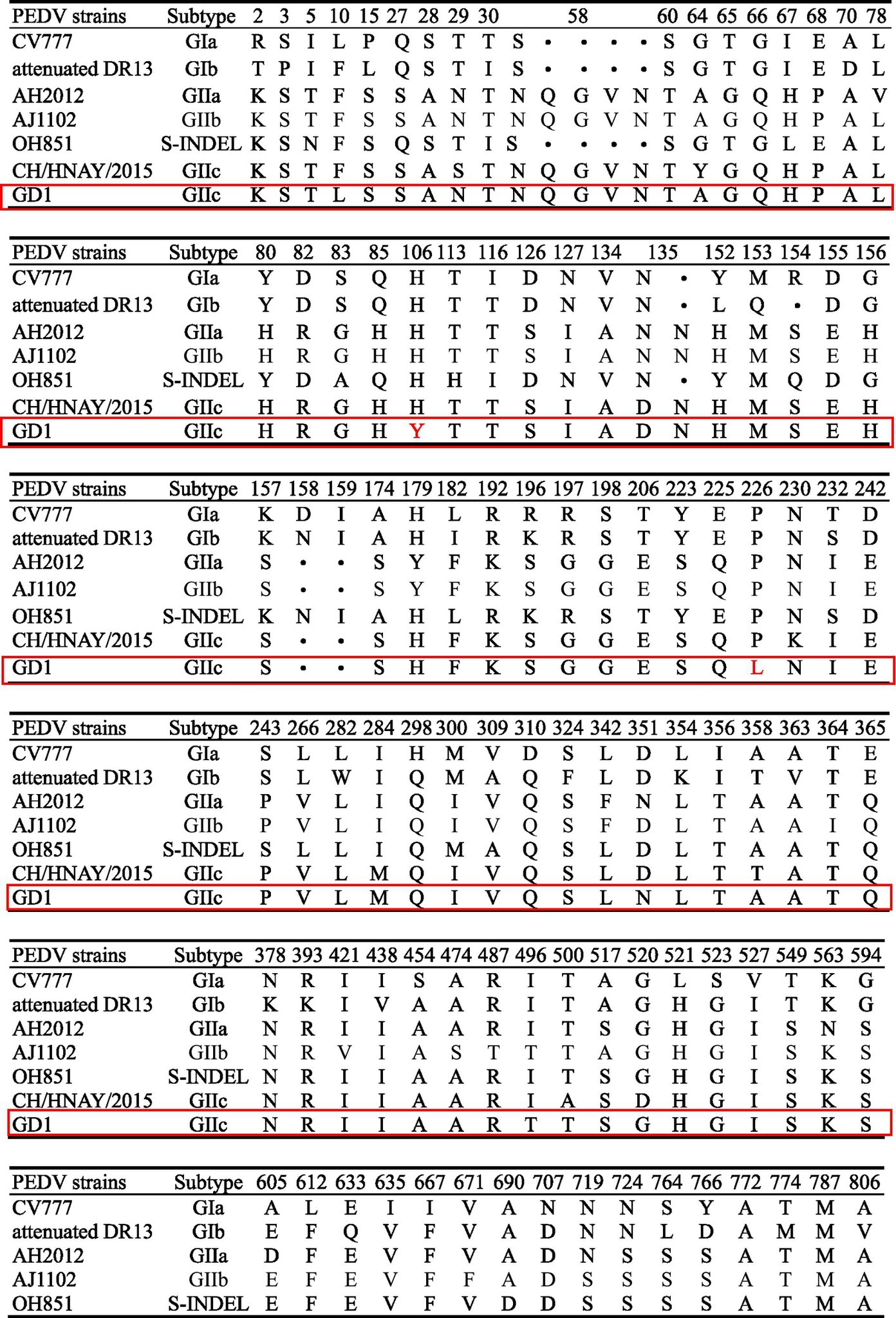
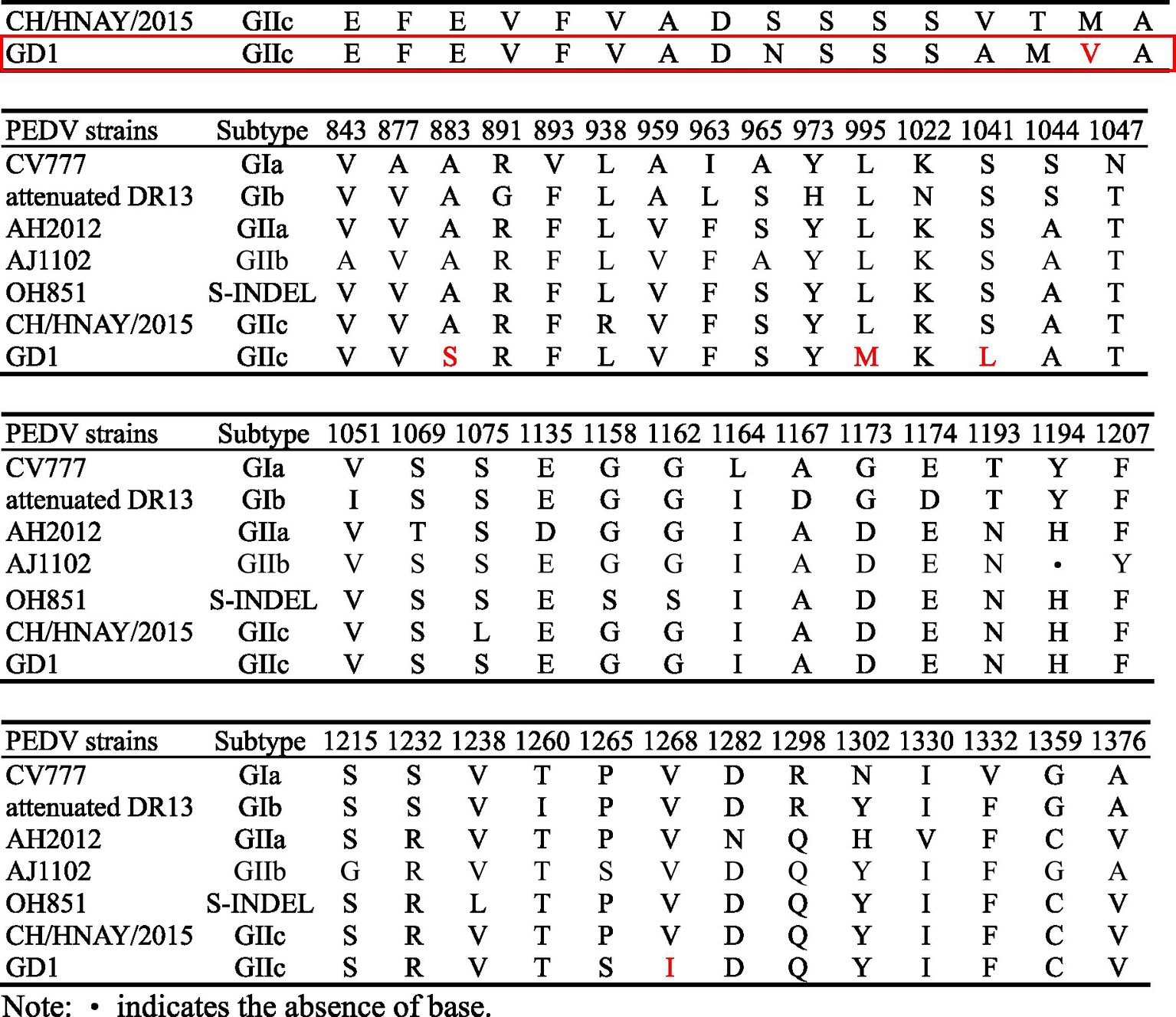

### 3.4 Recombination of PEDV GD1

**Figure 5:**
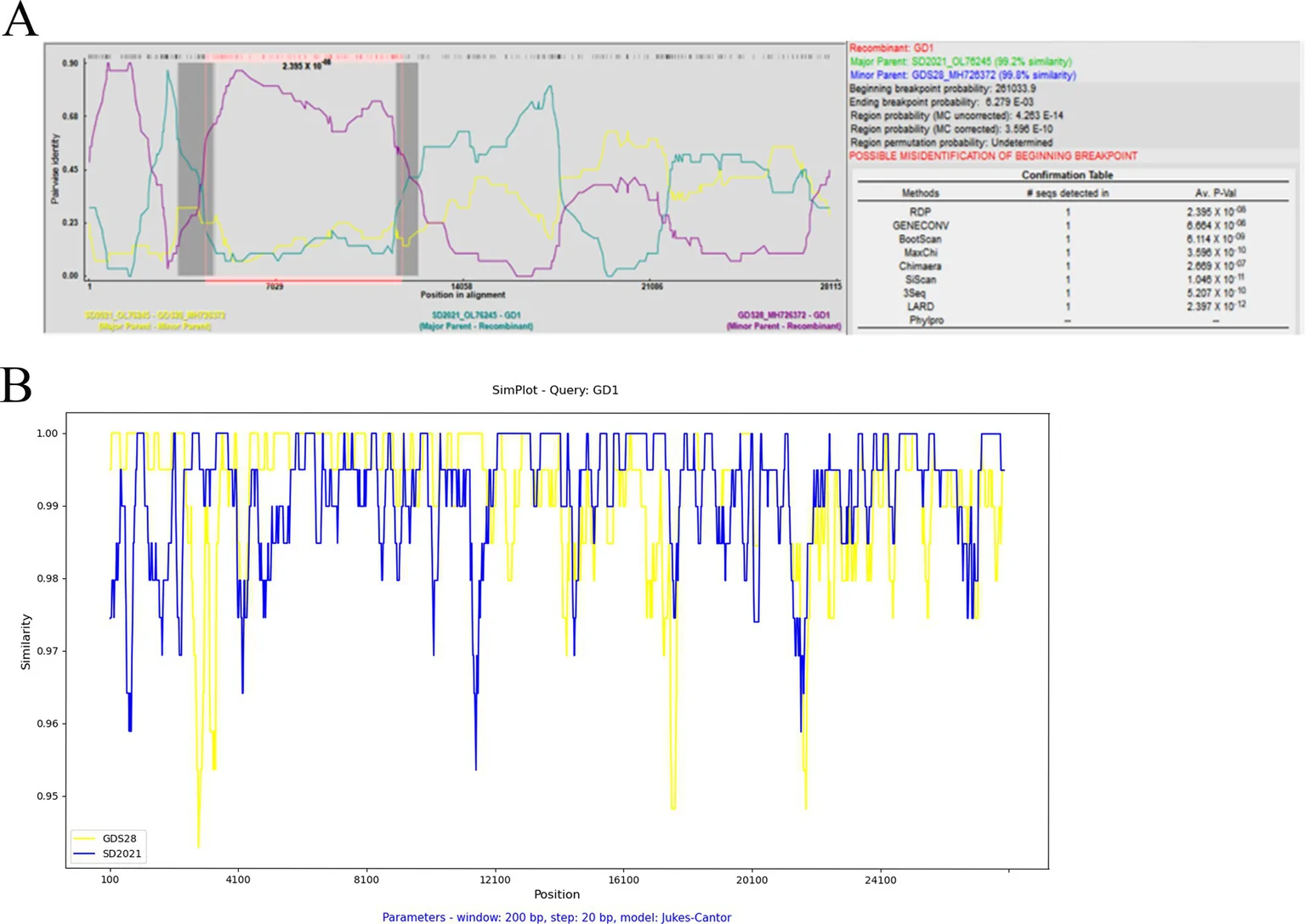
Recombination analysis of the PEDV GD1 strain. **(A)** Schematic diagram of putative recombination events detected by RDP4. **(B)** SimPlot analysis, SD2021 (blue) and GDS28 (yellow) were identified as putative parental strains.

To determine whether recombination events occurred in the isolated GD1 strain, recombination analysis was performed using the RDP4 software, followed by further sequence examination with Simplot. The results indicated that the GD1 strain originated from a recombination between the major parental strain SD2021 and the minor parental strain GDS28. The major parental strain SD2021 showed 99.2% similarity, and the minor parental strain GDS28 showed 99.8% similarity. Notably, this recombination event was consistently supported by 8 out of the 9 detection algorithms; the highest confidence was observed in the LARD (*p* = 2.397 × 10^−12^) and SiScan (*p* = 1.046 × 10^−11^) methods. The potential recombination breakpoints were located within the ORF1a region (nt 4,380–11,796) (Figure 5).

### 3.5 Pathogenicity analysis of PEDV GD1

**Figure 6:**
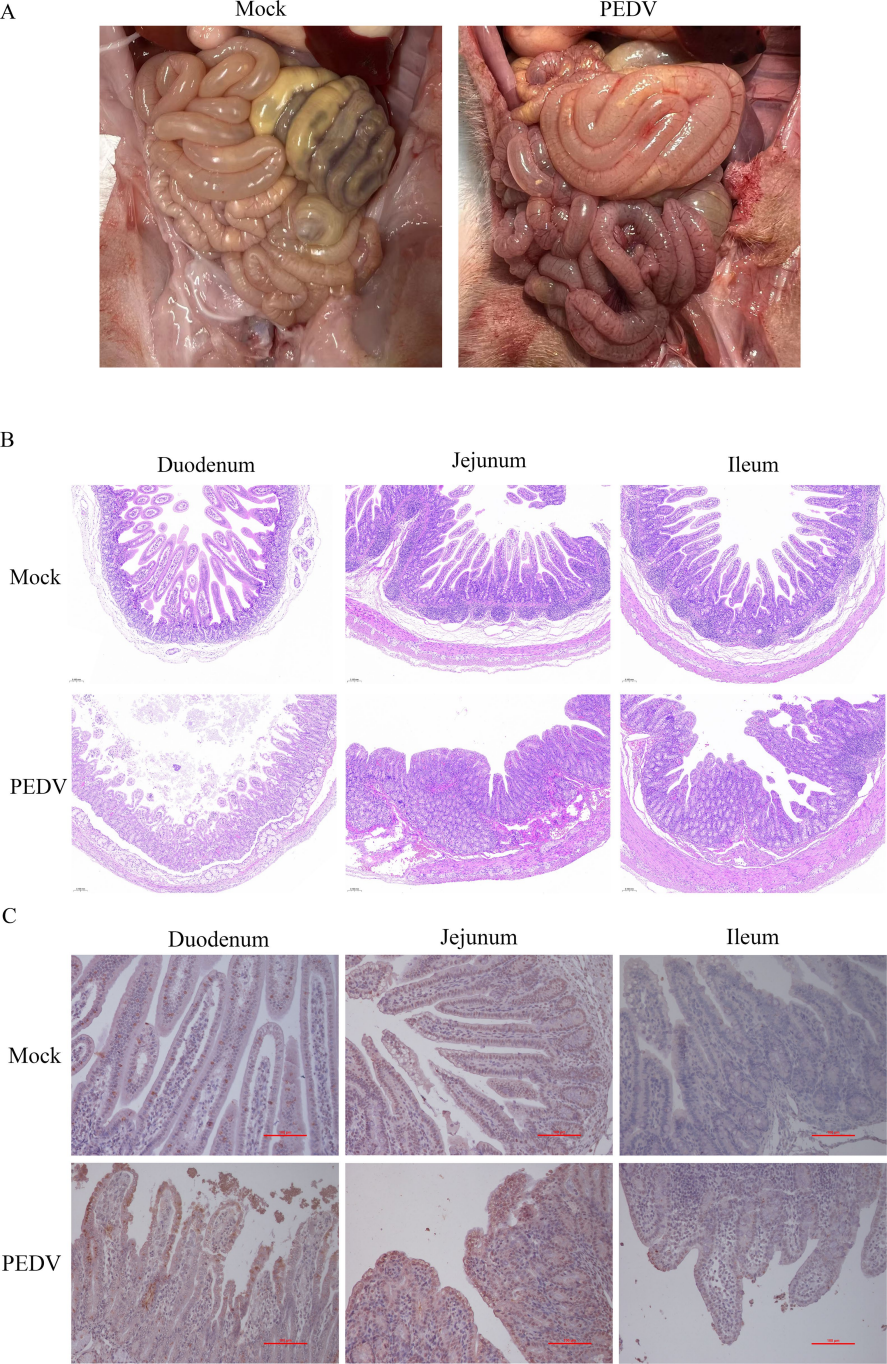
Pathogenicity analysis of PEDV GD1 strain. **(A)** Intestinal pathological lesion of PEDV GD1-challenged piglets at 48 hpi. **(B)** Histopathology examinations of duodenum, jejunum, and ileum from PEDV GD1-challenged piglets and control piglets at 48 hpi (hematoxylin and eosin staining) (100×). **(C)** Immunohistochemistry of duodenum, jejunum, and ileum from PEDV GD1-challenged piglets and control piglets at 48 hpi (200×).

Newborn piglets were used in challenge experiment to evaluate the pathogenicity of the PEDV GD1 strain. The symptoms of poor appetite, lethargy and yellow liquid feces were observed in challenged piglets at 12 h post inoculation (hpi). By 24 hpi, all three piglets developed watery diarrhea, severe emaciation, and even were unable to stand. In contrast, no obvious clinical symptoms were observed in the control piglets. At 48 hpi, the piglets were euthanized and then pathological changes were examined after necropsy. No pathological changes were found in the intestines of the piglets in the control group, while the challenged piglets displayed markedly swollen, hemorrhagic, and thin-walled intestines filled with abundant yellow fluid (Figure 6A). Further histopathological examination (H&E staining) demonstrated typical intestinal lesions and obvious microscopic changes. The villi in the duodenum, jejunum, and ileum of the challenged piglets were blunted, shortened, or even detached (Figure 6B). In addition, PEDV antigen in the small intestine were detected using immunohistochemistry (IHC). The results showed that PEDV antigen was present in whole intestines, including duodenum, jejunum, and ileum, and mainly distributed in the villus epithelial cells (Figure 6C).

## 4 Discussion

As a pathogen that seriously threatens the global swine industry, PEDV can cause severe diarrhea in piglets, resulting in enormous economic losses. Since the first case of PEDV was discovered in Europe in the early 1970s, the virus has been spreading worldwide and continuously mutating. In 2010, G2 genotype, a new variant of PEDV, was first discovered in central China. In 2014, S-INDEL, another genotype of PEDV, emerged in the USA. Since then, more and more variant PEDV strains have been detected worldwide. Notably, the GIIc lineage has become increasingly predominant in China in recent years, characterized by frequent recombination events and ongoing antigenic drift that compromise vaccine-induced protection (27, 34). The prevention and control of PEDV mainly rely on vaccines, but the continuous mutation of the virus undermines the efficacy of traditional vaccines (such as those developed based on classical strains like CV777) against emerging variant strains (35). The mismatch between the vaccine strain and the prevalent strain has become the main problem currently faced in prevention and control (36). Therefore, isolating the current prevalent strain, tracking their evolutionary characteristics, and assessing their pathogenicity are of vital importance for stockpiling vaccine candidate strains to prevent and control this disease. In this study, we successfully isolated a PEDV mutant strain from the intestines of diarrheic piglets on a farm in Guangdong Province, China, designated GD1, and evaluated its genetic evolution characteristics and pathogenicity.

Both sequence comparison and phylogenetic analysis based on the complete genome and S gene sequences revealed that the isolated GD1 strain belongs to the GIIc genotype. This classification aligns with the current epidemiological landscape of PEDV in Guangdong Province, where GIIc has been reported as the dominant lineage (25). Notably, GIIc strains exhibit distinct molecular characteristics. A comprehensive analysis of the PEDV S genes isolated in China from 2020 to 2023 concluded that the S gene of the GIIc strain has two characteristic amino acid substitutions, N139D and I289M, which does not exist in other subtypes (23). However, in the GD1 strain, only the N139D substitution was identified, while I289M was not detected. This deviation from the reported GIIc signature suggests that GD1 may represent a minor genetic variant within the GIIc lineage or that the I289M substitution is not universally conserved among all GIIc strains. This finding underscores the ongoing genetic diversification of the GIIc lineage. Furthermore, the GII subtype is often involved in recombination events, which have been identified across multiple genomic regions, including ORF1a, ORF1b, and the S gene (37). These recombination events contribute substantially to the emergence of novel variants and complicate disease control efforts. Therefore, continuous surveillance of this genetically distinct subgroup should be strengthened to ensure the vaccine efficacy.

The PEDV S protein is a critical viral protein responsible for receptor binding and mediating viral entry into host cells. It is also the most variable protein, and mutations in the S protein are a major driver of alterations in viral virulence and immune evasion (38, 39). Therefore, investigating the S protein not only contributes to a deeper understanding of PEDV pathogenesis but also provides a crucial foundation for developing novel vaccines and diagnostic reagents. The S protein comprises two subunits, S1 and S2. The S1 subunit is responsible for recognizing and binding to receptors on the host cell surface, whereas the S2 subunit mediates the fusion of the viral envelope with the host cell membrane (40, 41). In addition to its essential role in viral infection, the S protein exhibits strong antigenicity and induces the production of neutralizing antibodies. Several neutralizing epitopes have been identified to date, including COE (aa 499–638), SE16 (aa 722–731), SS2 (aa 748–755), SS6 (aa 764–771), and 2C10 (aa 1,368–1,374) (42–44). Mutations within these epitopes can alter antigenicity and facilitate immune escape, thereby compromising vaccine efficacy (45–47). Therefore, monitoring amino acid variations in these neutralizing epitopes is critical for evaluating the protective potential of current vaccines against emerging PEDV variants. In this study, amino acid sequence analysis of the S protein of the GD1 strain revealed multiple amino acid mutations in several neutralizing epitopes compared with the classical strain CV777. Notably, nine amino acid substitutions (A517S, L521H, S523G, V527I, T549S, G594S, A605E, L612F and I635V) were identified within the COE domain. Among these, eight substitutions (L521H, S523G, V527I, T549S, G594S, A605E, L612F and I635V) are considered to be common in GII subtype strains. It has been proposed that some substitutions (L521H, S523G, V527I, T549S, G594S, A605E and L612F) may help explain why traditional inactivated and attenuated vaccines fail to provide effective protection against epidemics caused by GII genogroup strains (20). The A517S mutation, however, was present in only a small number of GII subtype strains (22), suggesting it may represent a less frequent variant within the GII lineage. In addition, in this study, we identified seven unique amino acid mutations (H106Y, P226L, M787V, A883S, L995M, L1041S and V1268I) in S protein of GD1 strain. Although the functional consequences of these unique mutations remain to be characterized, their locations within functionally important domains suggest they may influence viral structure, stability, or immune recognition. For instance, P226L is located within the S1 domain, a region critical for receptor recognition, leading us to hypothesize that this substitution may alter receptor binding affinity or specificity. However, the functional significance of these mutations was not experimentally validated in this study, and no neutralization assays, receptor binding assays, or reverse genetics experiments were performed. Accordingly, the proposed roles of these mutations in immune escape or virulence remain hypothetical and warrant further investigation. Nonetheless, the spatial clustering of these mutations within functionally important domains (COE and S1) provides a molecular basis for prioritizing future functional studies. Notably, previous studies have provided valuable references for mapping mutations onto three-dimensional structural models of the PEDV S protein using tools such as AlphaFold or PyMOL (48, 49). Such structural analyses could offer insights into the potential impact of these mutations on antigenic variation and receptor interactions, thereby guiding the design of targeted functional experiments in the future.

Recombination is a major evolutionary mechanism driving the genetic diversity and adaptation of RNA viruses, especially coronaviruses, including PEDV (8, 50). GIIc subtype has been identified as the predominant subtype circulating in China (51), and accumulating evidence has shown that GIIc subtypes are frequently involved in recombination events (27, 50, 52). In this study, recombination analysis using RDP4 revealed that GD1 likely originated from a recombination between the major parental strain SD2021 and the minor parental strain GDS28, with the breakpoint mapping to 4,380–11,796 nt within the ORF1a region. The recombination event was supported by all eight algorithms with high statistical confidence (*p* < 0.01), consistent with the methodological standards established in previous PEDV recombination studies (53). GDS28 has been previously identified as a minor parent in the recombination event giving rise to HNAY strain (54), a recombinant PEDV isolated from Henan Province in 2016. Both Henan and Guangdong are major pig-raising provinces in China, and were identified as primary hubs for the spread of PEDV. The frequent inter-provincial pig trade between them may have facilitated the spread of recombinant strains such as GDS28 from central China to southern China (55). Moreover, in this study, the recombination breakpoint in GD1 lies within ORF1a. A recent study reported a highly virulent recombinant GIIc strain (CHjx2025) isolated from Jiangxi Province with a recombination breakpoint mapped within the ORF1a region at nucleotide 11,201 (27), further confirming that ORF1a recombination is an emerging evolutionary feature among GIIc strains circulating in southern China. ORF1a encodes the viral replicase complex, including nonstructural proteins (nsps) involved in RNA synthesis and processing. Although the precise functional impact of recombination in this region remains unclear, previous studies have suggested that nonstructural proteins encoded by ORF1a play critical roles in viral replication and innate immune modulation. Variations in this region may influence replication efficiency and pathogenicity, as suggested by studies showing that nsp1 and nsp14 are involved in the regulation of innate immunity and viral replication (12, 56, 57). Our results underscore the importance of continuous surveillance for recombinant strains, as recombination may accelerate the emergence of variants with altered virulence or antigenicity.

Numerous studies have reported that GIIc subtype strains exhibit high pathogenicity (51, 58, 59). In the present study, the GD1 strain demonstrated strong infectivity in Vero cells, with obvious cell fusion and syncytial formation observed in infected cells. Animal challenge experiments revealed that newborn piglets infected with the GD1 strain developed distinct clinical symptoms within 12 hpi, including poor appetite, lethargy, watery diarrhea, and severe emaciation, whereas piglets in the control group remained normal. Necropsy revealed obvious intestinal lesions in infected piglets. Histopathological examination showed atrophy, shortening, and even shedding of intestinal villi in the duodenum, jejunum, and ileum, which were consistent with the typical lesions caused by PEDV. These findings indicate that the GD1 strain, like other GIIc strains, retains high pathogenicity. Immunohistochemistry further confirmed that PEDV antigen was predominantly distributed in the villous epithelial cells of the small intestine, which was consistent with the findings of Li et al. (54). However, it should be noted that the controlled laboratory challenge conditions may not fully reflect the complexity of natural infection, where factors such as maternal immunity, co-infections, and environmental conditions modulate disease outcomes. It should also be acknowledged that this pathogenicity assessment was conducted with a limited sample size (*n* = 3 per group), which precludes formal statistical analysis. In addition, quantitative parameters such as body weight changes, diarrhea scores, and fecal viral RNA loads were not measured in this preliminary study. Despite these limitations, the consistent and pronounced clinical signs and pathological lesions observed across all challenged piglets provide clear evidence of the high virulence of GD1. Nevertheless, the virulence of the GD1 strain may be associated with multiple factors. In-depth investigation of the factors influencing its virulence is therefore warranted. Such studies will facilitate the development of better-matched vaccines for effective disease control.

In conclusion, the PEDV GD1 strain isolated in this study from a pig farm in Guangdong Province, China, belongs to the GIIc genotype, with multiple amino acid substitutions identified in the neutralizing epitopes of S protein. GD1 strain is a recombinant strain, with the potential recombination breakpoint located in the ORF1a region. The recombinant strain exhibits both high adaptability to Vero cells and strong pathogenicity in newborn piglets. In addition, several unique amino acid substitutions were identified in the GD1 strain, although their functional roles in viral pathogenicity remain to be elucidated. Collectively, these findings underscore the need for enhanced continuous surveillance and precise tracking of viral evolution to facilitate the development of better-matched vaccines and improve disease control strategies.

## Data Availability

The datasets presented in this study can be found in online repositories. The names of the repository/repositories and accession number(s) can be found at: https://www.ncbi.nlm.nih.gov/nuccore/PX146708.

## Footnotes

1
https://www.ncbi.nlm.nih.gov/genbank/

## References

[1] Chen X Zhang XX Li C Wang H Wang H Meng XZ . Epidemiology of porcine epidemic diarrhea virus among Chinese pig populations: a meta-analysis. Microb Pathog. (2019) 129:43–9. doi: 10.1016/j.micpath.2019.01.017, 30682525

[2] Li M Pan Y Xi Y Wang M Zeng Q. Insights and progress on epidemic characteristics, genotyping, and preventive measures of PEDV in China: a review. Microb Pathog. (2023) 181:106185. doi: 10.1016/j.micpath.2023.106185, 37269880

[3] Peng Q Fu P Zhou Y Lang Y Zhao S Wen Y . Phylogenetic analysis of porcine epidemic diarrhea virus (PEDV) during 2020–2022 and isolation of a variant recombinant PEDV strain. Int J Mol Sci. (2024) 25:10878. doi: 10.3390/ijms252010878, 39456662 PMC11507624

[4] Wang PH Li YQ Pan YQ Guo YY Guo F Shi RZ . The spike glycoprotein genes of porcine epidemic diarrhea viruses isolated in China. Vet Res. (2021) 52:87. doi: 10.1186/s13567-021-00954-6, 34130762 PMC8205199

[5] Stevenson GW Hoang H Schwartz KJ Burrough ER Sun D Madson D . Emergence of porcine epidemic diarrhea virus in the United States: clinical signs, lesions, and viral genomic sequences. J Vet Diagn Invest. (2013) 25:649–54. doi: 10.1177/1040638713501675, 23963154

[6] Li R Qiao S Yang Y Guo J Xie S Zhou E . Genome sequencing and analysis of a novel recombinant porcine epidemic diarrhea virus strain from Henan, China. Virus Genes. (2016) 52:91–8. doi: 10.1007/s11262-015-1254-1, 26743534 PMC7089116

[7] Li K Song D Zhang F Gong W Guo N Li A . Complete genome sequence of a recombinant porcine epidemic diarrhea virus strain, CH/JXJA/2017, isolated in Jiangxi, China, in 2017. Genome Announc. (2018) 6:e01590-17. doi: 10.1128/genomeA.01590-17, 29439052 PMC5805890

[8] Zhang H Zou C Peng O Ashraf U Xu Q Gong L . Global dynamics of porcine enteric coronavirus pedv epidemiology, evolution, and transmission. Mol Biol Evol. (2023) 40:msad052. doi: 10.1093/molbev/msad052, 36869744 PMC10027654

[9] Turlewicz-Podbielska H Pomorska-Mól M. Porcine coronaviruses: overview of the state of the art. Virol Sin. (2021) 36:833–51. doi: 10.1007/s12250-021-00364-0, 33723809 PMC7959302

[10] Kocherhans R Bridgen A Ackermann M Tobler K. Completion of the porcine epidemic diarrhoea coronavirus (PEDV) genome sequence. Virus Genes. (2001) 23:137–44. doi: 10.1023/a:1011831902219, 11724265 PMC7089135

[11] Lin F Zhang H Li L Yang Y Zou X Chen J . PEDV: insights and advances into types, function, structure, and receptor recognition. Viruses. (2022) 14:1744. doi: 10.3390/v14081744, 36016366 PMC9416423

[12] Li Z Ma Z Li Y Gao S Xiao S. Porcine epidemic diarrhea virus: molecular mechanisms of attenuation and vaccines. Microb Pathog. (2020) 149:104553. doi: 10.1016/j.micpath.2020.104553, 33011361 PMC7527827

[13] Wang K Lu W Chen J Xie S Shi H Hsu H . PEDV ORF3 encodes an ion channel protein and regulates virus production. FEBS Lett. (2012) 586:384–91. doi: 10.1016/j.febslet.2012.01.005 22245155 PMC7094521

[14] Lu Y Huang W Lu Z Zeng D Yu K Bai J . Genetic characteristics associated with the virulence of porcine epidemic diarrhea virus (PEDV) with a naturally occurring truncated ORF3 gene. Vet Res. (2024) 55:123. doi: 10.1186/s13567-024-01384-w, 39334484 PMC11437794

[15] Qiu Y Sun Y Zheng X Gong L Yang L Xiang B. Identification of host proteins interacting with the E protein of porcine epidemic diarrhea virus. Front Microbiol. (2024) 15:1380578. doi: 10.3389/fmicb.2024.1380578, 38577683 PMC10994376

[16] Liu X Zhang M Luo Y Yan S Xu H Yao Y . Surface expression of PEDV nucleocapsid protein facilitates NK cell-mediated ADCC via anti-N nonneutralizing antibody. Vet Microbiol. (2025) 305:110518. doi: 10.1016/j.vetmic.2025.110518, 40233683

[17] Chang CY Cheng IC Chang YC Tsai PS Lai SY Huang YL . Identification of neutralizing monoclonal antibodies targeting novel conformational epitopes of the porcine epidemic diarrhoea virus spike protein. Sci Rep. (2019) 9:2529. doi: 10.1038/s41598-019-39844-5, 30792462 PMC6385244

[18] Xu H Wu H Min J Fu N Shi Y Zhou P . Effective control of the emerging PEDV G2-c variant with an inactivated autogenous vaccine. Front Vet Sci. (2025) 12:1697499. doi: 10.3389/fvets.2025.1697499, 41164227 PMC12559854

[19] Li Y Yang S Qian J Liu S Li Y Song X . Molecular characteristics of the immune escape of coronavirus PEDV under the pressure of vaccine immunity. J Virol. (2025) 99:e0219324. doi: 10.1128/jvi.02193-24, 40237499 PMC12090811

[20] Guo J Fang L Ye X Chen J Xu S Zhu X . Evolutionary and genotypic analyses of global porcine epidemic diarrhea virus strains. Transbound Emerg Dis. (2019) 66:111–8. doi: 10.1111/tbed.12991, 30102851 PMC7168555

[21] Li C Wu Q Song H Lu H Yang K Liu Z . Elucidating the biological characteristics and pathogenicity of the highly virulent G2a porcine epidemic diarrhea virus. J Gen Virol. (2024) 105:e001953. doi: 10.1099/jgv.0.001953, 38270573

[22] Fu Y Wang Y Dai L Cheng B Xiao S Yin Y. Evolutionary dynamics and antigenic diversity of porcine epidemic diarrhea virus (PEDV) in China: phylogenetic and recombination analyses based on large-scale S gene sequences. BMC Vet Res. (2025) 21:426. doi: 10.1186/s12917-025-04873-y, 40604964 PMC12220379

[23] Wang Y Qian J Li Y Wang D Song X Tian S . Genetic characterization and phylogenetic analysis of the S genes of porcine epidemic diarrhea virus isolates from China from 2020 to 2023. Arch Virol. (2024) 169:180. doi: 10.1007/s00705-024-06109-0, 39150572

[24] Lu X Chen C Wang Z Zhang A. Isolation and characterization of porcine epidemic diarrhea virus G2c strains circulating in China from 2021 to 2024. Vet Sci. (2025) 12:444. doi: 10.3390/vetsci12050444, 40431537 PMC12116066

[25] Huang L Yan L Zeng M Yao J Hu J Zhong W . G2c-lineage dominance and S1 epitope-glycan drift of porcine epidemic diarrhea virus in Guangdong Province, China, 2022–2024. Vet Sci. (2025) 12:1056. doi: 10.3390/vetsci12111056, 41295693 PMC12656786

[26] Cao Y Cui A Chen X Cheng Y Tang R Zeng H . Genetic diversity, recombination, and pathogenicity of porcine epidemic diarrhea virus strains circulating in China during 2023–2024. Transbound Emerg Dis. (2026) 2026:1340053. doi: 10.1155/tbed/1340053, 42164053 PMC13184637

[27] Fan J Liu Y Fu W Chen L Li Y Wang Z . Isolation and pathogenicity of a highly virulent recombinant GIIc subtype PEDV strain. BMC Vet Res. (2026). [ahead-of-print]. doi: 10.1186/s12917-026-05714-2 42443885

[28] Xu J Fu N Liu Z Chen M Ma G Li H . A novel inactivated vaccine based on an emerging PEDV GIIc variant provides cross-protection against heterologous GII strains. Vaccine. (2026) 14:151. doi: 10.3390/vaccines14020151, 41746072 PMC12945236

[29] Ji Z Shi D Shi H Wang X Chen J Liu J . A porcine epidemic diarrhea virus strain with distinct characteristics of four amino acid insertion in the COE region of spike protein. Vet Microbiol. (2021) 253:108955. doi: 10.1016/j.vetmic.2020.108955, 33373882 PMC7733691

[30] Wang S Hu X Xiong T Cao L Zhang X Song Z. Isolation of porcine epidemic diarrhea virus strain CHCQ-2023 from Chongqing Province and analysis of S gene recombination. BMC Vet Res. (2024) 20:565. doi: 10.1186/s12917-024-04390-4, 39695646 PMC11657806

[31] Li YY Chen KF Fan CH Li HX Zhen HQ Zhu YQ . Clade-specific recombination and mutations define the emergence of porcine epidemic diarrhea virus S-INDEL lineages. Animals (Basel). (2025) 15:2312. doi: 10.3390/ani15152312, 40805110 PMC12345761

[32] Wen F Yang J Li A Gong Z Yang L Cheng Q . Genetic characterization and phylogenetic analysis of porcine epidemic diarrhea virus in Guangdong, China, between 2018 and 2019. PLoS One. (2021) 16:e0253622. doi: 10.1371/journal.pone.0253622, 34166425 PMC8224968

[33] Sun Y Gong T Wu D Feng Y Gao Q Xing J . Isolation, identification, and pathogenicity of porcine epidemic diarrhea virus. Front Microbiol. (2023) 14:1273589. doi: 10.3389/fmicb.2023.1273589, 37904874 PMC10613466

[34] Gao M Liu Y Xu X Chen P Gong L Wang H . Temporal evolutionary dynamics of porcine epidemic diarrhea virus in China from 2013 to 2023. Infect Genet Evol. (2026) 141:105938. doi: 10.1016/j.meegid.2026.105938, 41962875

[35] Sun RQ Cai RJ Chen YQ Liang PS Chen DK Song CX. Outbreak of porcine epidemic diarrhea in suckling piglets, China. Emerg Infect Dis. (2012) 18:161–3. doi: 10.3201/eid1801.111259, 22261231 PMC3381683

[36] Jung K Saif LJ Wang Q. Porcine epidemic diarrhea virus (PEDV): an update on etiology, transmission, pathogenesis, and prevention and control. Virus Res. (2020) 286:198045. doi: 10.1016/j.virusres.2020.198045, 32502552 PMC7266596

[37] Yan N Xu J Li Y Fan S Qiu S Huang L . A recombinant porcine epidemic diarrhea virus with multiple S2 subunit mutations from China: isolation, genetic characterization, and pathogenicity analysis. Microorganisms. (2026) 14:242. doi: 10.3390/microorganisms14010242, 41597759 PMC12844477

[38] Liu H Yin X Tian H Qiu Y Wang Z Chen J . The S protein of a novel recombinant PEDV strain promotes the infectivity and pathogenicity of PEDV in mid-West China. Transbound Emerg Dis. (2022) 69:3704–23. doi: 10.1111/tbed.14740, 36251324

[39] Li W Hangalapura BN van den Elzen P van den Born E van Kuppeveld FJM Rottier PJM . Spike gene variability in porcine epidemic diarrhea virus as a determinant for virulence. J Virol. (2025) 99:e0216524. doi: 10.1128/jvi.02165-24, 40001283 PMC11915861

[40] Li F. Structure, function, and evolution of coronavirus spike proteins. Annu Rev Virol. (2016) 3:237–61. doi: 10.1146/annurev-virology-110615-042301, 27578435 PMC5457962

[41] Luo H Liang Z Lin J Wang Y Liu Y Mei K . Research progress of porcine epidemic diarrhea virus S protein. Front Microbiol. (2024) 15:1396894. doi: 10.3389/fmicb.2024.1396894 38873162 PMC11169810

[42] Chang SH Bae JL Kang TJ Kim J Chung GH Lim CW . Identification of the epitope region capable of inducing neutralizing antibodies against the porcine epidemic diarrhea virus. Mol Cells. (2002) 14:295–9. doi: 10.1016/S1016-8478(23)15106-5, 12442904

[43] Sun D Feng L Shi H Chen J Cui X Chen H . Identification of two novel B cell epitopes on porcine epidemic diarrhea virus spike protein. Vet Microbiol. (2008) 131:73–81. doi: 10.1016/j.vetmic.2008.02.022, 18400422 PMC7117171

[44] Cruz DJ Kim CJ Shin HJ. The GPRLQPY motif located at the carboxy-terminal of the spike protein induces antibodies that neutralize porcine epidemic diarrhea virus. Virus Res. (2008) 132:192–6. doi: 10.1016/j.virusres.2007.10.015, 18067984 PMC7114191

[45] Li C Li W Lucio de Esesarte E Guo H van den Elzen P Aarts E . Cell attachment domains of the porcine epidemic diarrhea virus spike protein are key targets of neutralizing antibodies. J Virol. (2017) 91:e00273-17. doi: 10.1128/JVI.00273-17, 28381581 PMC5446644

[46] Liu J Hu G Liu S Ren G Gao L Zhao Z . Evaluating passive immunity in piglets from sows vaccinated with a PEDV S protein subunit vaccine. Front Cell Infect Microbiol. (2024) 14:1498610. doi: 10.3389/fcimb.2024.1498610, 39963235 PMC11831279

[47] Hu G Luo X Liao J Zou C Huang Y Geng R . Neutralizing antibody levels as a key factor in determining the immunogenic efficacy of the novel PEDV alpha coronavirus vaccine. Vet Q. (2025) 45:1–20. doi: 10.1080/01652176.2025.2509506, 40432512 PMC12120861

[48] Wrapp D McLellan JS. The 3.1-angstrom cryo-electron microscopy structure of the porcine epidemic diarrhea virus spike protein in the prefusion conformation. J Virol. (2019) 93:e00923-19. doi: 10.1128/JVI.00923-19, 31534041 PMC6854500

[49] Ma Z Li Z Li Y Zhao X Zheng C Li Y . Changes in the motifs in the D0 and SD2 domains of the S protein drive the evolution of virulence in enteric coronavirus porcine epidemic diarrhea virus. J Virol. (2025) 99:e0209224. doi: 10.1128/jvi.02092-24, 40035514 PMC11998522

[50] Tian Y Yang X Li H Ma B Guan R Yang J . Molecular characterization of porcine epidemic diarrhea virus associated with outbreaks in Southwest China during 2014–2018. Transbound Emerg Dis. (2021) 68:3482–97. doi: 10.1111/tbed.13953, 33306274

[51] Li YY Fan CH Li HX Zhen HQ Zhu YQ Wang S . Emergence of a highly virulent porcine epidemic diarrhea virus (PEDV) G2c subtype in China: isolation, genetic and pathogenic characterization, and cross-neutralizing antibody response. Transbound Emerg Dis. (2026) 2026:3811264. doi: 10.1155/tbed/3811264, 41710531 PMC12910253

[52] Shi K Li B Shi Y Feng S Yin Y Long F . Phylogenetic and evolutionary analysis of porcine epidemic diarrhea virus in Guangxi Province, China, during 2020 and 2024. Viruses. (2024) 16:1126. doi: 10.3390/v16071126, 39066288 PMC11281377

[53] Martin DP Murrell B Golden M Khoosal A Muhire B. RDP4: detection and analysis of recombination patterns in virus genomes. Virus Evol. (2015) 1:vev003. doi: 10.1093/ve/vev003, 27774277 PMC5014473

[54] Li D Li Y Liu Y Chen Y Jiao W Feng H . Isolation and identification of a recombinant porcine epidemic diarrhea virus with a novel insertion in S1 domain. Front Microbiol. (2021) 12:667084. doi: 10.3389/fmicb.2021.667084, 33959119 PMC8093569

[55] He WT Bollen N Xu Y Zhao J Dellicour S Yan Z . Phylogeography reveals association between swine trade and the spread of porcine epidemic diarrhea virus in China and across the world. Mol Biol Evol. (2022) 39:msab364. doi: 10.1093/molbev/msab364, 34951645 PMC8826572

[56] Zhang Y Chen Y Zhou J Wang X Ma L Li J . Porcine epidemic diarrhea virus: an updated overview of virus epidemiology, virulence variation patterns and virus-host interactions. Viruses. (2022) 14:2434. doi: 10.3390/v14112434, 36366532 PMC9695474

[57] Zhang J Yuan S Peng Q Ding Z Hao W Peng G . Porcine epidemic diarrhea virus nsp7 inhibits interferon-induced JAK-STAT signaling through sequestering the interaction between KPNA1 and STAT1. J Virol. (2022) 96:e0040022. doi: 10.1128/jvi.00400-22, 35442061 PMC9093119

[58] Li X Li Y Huang J Yao Y Zhao W Zhang Y . Isolation and oral immunogenicity assessment of porcine epidemic diarrhea virus NH-TA2020 strain: one of the predominant strains circulating in China from 2017 to 2021. Virol Sin. (2022) 37:646–55. doi: 10.1016/j.virs.2022.08.002, 35961502 PMC9583181

[59] Pan S Zou J Mou C Wang Z Liu H Fu L . Enhancing the virulence of G2a subtype PEDV by chimeric expression of G2c subtype S gene. Vet Microbiol. (2025) 310:110728. doi: 10.1016/j.vetmic.2025.110728, 40976147

